# Acquisition of Higher-Order Cognitive Skills (HOCS) Using the Flipped Classroom Model: A Quasi-Experimental Study

**DOI:** 10.7759/cureus.24249

**Published:** 2022-04-18

**Authors:** Swapnil Paralikar, Chinmay J Shah, Anuradha Joshi, Rajesh Kathrotia

**Affiliations:** 1 Physiology, Government Medical College, Bhavnagar, Bhavnagar, IND; 2 Pharmacology, Pramukhswami Medical College, Anand, IND; 3 Physiology, All India Institute of Medical Sciences, Rajkot, Rajkot, IND

**Keywords:** student centered learning, andragogy, medical education, higher order cognitive skills, flipped classroom

## Abstract

Introduction

Flipped classroom refers to a teaching model where the lecture and homework elements of the class are reversed. Students develop knowledge and understanding during the pre-class session, and use the in-class time for active learning such as small group discussions, wherein they are expected to develop the skills of problem-solving (application) and critical thinking (analysis). An educational psychologist, Benjamin Bloom, proposed Bloom’s Taxonomy for the cognitive domain. According to this taxonomy, knowledge, and understanding have been considered to be lower-order cognitive skills (LOCS), while application (problem-solving), analysis (critical thinking), synthesis, and evaluation have been considered higher-order cognitive skills (HOCS). Only one study has reported that flipped classroom approach helps in the acquisition of HOCS such as application, analysis, synthesis, and evaluation. The present study aims to compare the flipped classroom model with the traditional lecture model and focuses particularly on the acquisition of HOCS such as application and analysis, by assessing the students using multiple-choice questions based on clinical vignettes.

Methods

The study was conducted in the Department of Physiology at Government Medical College, Bhavnagar. A total of 102 students in two groups, Group A (n=52) and Group B (n=50), participated in all the phases of the study. In the flipped classroom group, students watched the pre-recorded videos and studied standard textbooks, followed by a group discussion in the class. The traditional Lecture group attended the didactic lecture only. Topic I was taught to Group A using the flipped classroom model and to Group B using the traditional didactic lecture approach. Topic II was taught to Group B by using the flipped classroom method and to Group A using the traditional didactic lecture.

Both groups of students were assessed after a gap of one week with multiple-choice questions based on clinical vignettes for checking the HOCS. These questions were designed based on Blooming Biology Tool (BBT).

The feedback collected responses regarding the learning experience, perceived value of the flipped classroom, the course materials, the teaching process, and the evaluation system.

Results

The students, who participated in the flipped classrooms, performed statistically better than those in the traditional lecture model (5.36 ± 1.69 vs. 4.94 ± 1.34) (p<0.05). The students strongly agreed that the flipped classroom method was an enjoyable way of learning: it enhanced their problem solving and analytical ability as well as developed their ability to work as team members and plan their own work. Students in both the flipped classrooms gave a similar perception indicated by the small effect size (r <0.3).

Conclusion

The pre-class time of the flipped classroom model helps students remember the facts and understand the concepts (both lower-order cognitive skills), and uses the valuable in-class time to master the application of the concepts and critically analyze them (application and analysis being higher-order cognitive skills).

## Introduction

Lectures are ubiquitous in medicine. Lecturing is the single most efficient way to impart a large amount of knowledge in a short period of time. However, lecturing is a 'double-edged sword.' The traditional lecture (called 'the sage on the stage' approach) does not help the students to gain the skills of problem-solving and critical thinking, which are essential for clinical reasoning [1].

The flipped classroom is a pedagogical tool in which students develop a basic understanding of the course material before the class, and use the in-class time for learner-centred activities such as group presentations and discussions. This new style of teaching puts learning back into the hands of the student. The teacher’s role is that of a facilitator (called 'the guide on the side' approach), wherein he steers the discussion and clarifies the queries of the student. The flipped classroom model was popularized by Eric Mazur, a physics professor at Harvard University. He claimed that learning gains were nearly tripled with this model. In 2008, Karl Fisch created a video called 'Shift Happens' and has been credited with the coining of the term 'flipped classroom' [2].

According to Edgar Dale’s Cone of Learning, we remember 10% of what we read, 20% of what we hear, 30% of what we see demonstrated, and 50% of what we see and hear - all forms of passive learning. However, we remember a startling 70% of what we say and 90% of the things we both say and do - forms of active learning. Dale thus concluded that active learning is more efficient than passive learning [3]. Studies in cognitive neuroscience have revealed that learning involves the active construction of meaning by the learner. Hence, a large body of evidence supports the paradigm that active learning is a much better form of learning than the passive mode of lectures [4]. The flipped classroom model embraces the active learning approach during the in-class discussion. Accordingly, a recent systematic review has reported that the flipped classroom model enhances the learning performance of students. It also reported that the students gave a positive perception of the flipped classroom model [5].

A number of forces have led to the emergence of learner-centred learning in medical education. Firstly, information is fast expanding; hence, the breadth and depth of the medical curriculum have grown tremendously over the past few decades. As a result, what is learnt during medical school is likely to lose its relevance during the practice years. Secondly, because medical knowledge is complex, it is a fundamental remit of medical training, that the student develops problem-solving and analytical skills. Thus, in order to succeed in the twenty-first century, it is essential that medical students gain competence in the 4Cs - communication, collaboration, critical thinking, and creativity. Critical thinking involves the ability to think for themselves, to critique, to filter, to prioritize, to apply, and to draw conclusions [6,7].

The educational psychologist, Benjamin Bloom, proposed Bloom’s Taxonomy for the cognitive domain. According to this taxonomy, knowledge, and understanding have been considered to be lower-order cognitive skills (LOCS), while application (problem-solving), analysis (critical thinking), synthesis, and evaluation have been considered higher-order cognitive skills (HOCS) [4]. Only one study has reported that flipped classroom helps in the acquisition of HOCS like application, analysis, synthesis, and evaluation [8]. 

The present study aims to answer the research question as to whether the flipped classroom model is superior to the traditional lecture for the acquisition of HOCS like application and analysis. Acquisition of these skills would be assessed by multiple-choice questions based on clinical vignettes.

## Materials and methods

Study population

The study was quasi-experimental and comparative in design. It was conducted in the Department of Physiology, Government Medical College, Bhavnagar. Phase I MBBS students (Bachelor of Medicine and Bachelor of Surgery) of the batch 2020-21, were divided into two groups based on their roll numbers; Group A consisted of roll numbers 1-100, while Group B consisted of roll numbers 101-200. The study was approved by the Ethics Committee of Government Medical College, Bhavnagar (1116/2021). Written informed consent was obtained from all the participants before the commencement of the study. Written informed consent was provided by 85 students in Group A and 84 students in Group B. However, the number of students who completed all the phases of the study as discussed below were: Group A (n=52) and Group B (n=50). Group A was taught Topic I (Cardiac Output) by the flipped classroom model. Group B was taught Topic II (Regulation of Blood Pressure) by the flipped classroom model. The same topic was taught to the other group by the traditional didactic lecture model.

Implementation of the study

Specific learning objectives for both topics were prepared. Videos to be watched by students online were prepared by the principal investigator. Each video was 8-10 minutes in length. Six such videos were prepared: 1) regulation of stroke volume 2) regulation of heart rate 3) baroreceptor mechanism for regulation of blood pressure 4) renin-angiotensin-aldosterone system 5) long term regulation of blood pressure 6) cardiovascular reflexes. Multiple choice questions with clinical vignettes were prepared based on the Blooming Biology Tool (BBT), to test the higher levels of the cognitive domain viz. application and analysis [9]. The specific learning objectives, the online videos and the items for assessment were validated by two external faculty members of physiology from different institutions. Two separate WhatsApp groups were created for sharing the videos and passing instructions to the students.

The chronology of the two classrooms is summarized in Table 1. Before the study, a pre-class test was conducted to determine the baseline knowledge of the students in both groups. The pre-test consisted of multiple-choice questions which were designed to test the students’ HOCS.

A Google Drive link of the videos was shared with the students in their respective WhatsApp groups, four to five days before the in-class session. They were instructed to watch the videos and read the topic in standard textbooks. Additionally, the instructor prepared and posted questions in the WhatsApp groups, which the students were supposed to solve.

Representative questions include: "1) Why does the traffic policeman faint if he stands in attention in hot weather for a long time? 2) Why do athletes have bradycardia? 3) Stress in the office makes the heart work harder. Explain".

During the in-class session, students were divided into groups of five to six members. Each group had a team leader, a scribe, a reporter and a timekeeper. Each group solved a set of questions during an assigned time period. The instructor solved any queries which arose in the discussion. At the end of the session, the instructor summarized the responses from all the groups, solved queries and provided feedback.

In the lecture-based classroom, the students attended a 60-min didactic lecture followed by 15 minutes of solving queries by the lecturer. The principal investigator, who had prepared the videos, provided direct instruction through the lectures as well. This ensured uniformity in the course content and delivery of the instruction. 

To investigate the students’ attitude towards the flipped classroom, all the students (n=102), were asked to fill out feedback questionnaires immediately after the class. The questionnaire was adapted from Paul Ramsden’s Course Experience Questionnaire and Biggs’ Study Process Questionnaire. The questionnaire contained questions regarding the learning experience, perceived value of the flipped classroom, course materials, teaching process and evaluation system. The questionnaire used a 4-point Likert scale (1=strongly disagree and 4=strongly agree). Prior to administering the questionnaire, the internal consistency of the questionnaire was calculated (Cronbach’s alpha=0.874).

**Table 1:** The chronology of the flipped classroom model and the lecture-based model

	Flipped Classroom Model	Lecture Based Model
Before class	Pre-test	
	Sharing of pre-recorded videos on the WhatsApp group; sharing of specific learning objectives and the questions to be solved by the students; reading of chapters on cardiac output/regulation of blood pressure from standard textbooks	Sharing of specific learning objectives with the students
During class	Formation of groups with observation of group dynamics (10 minutes) discussion in groups of six on the questions relating to the topic (60 minutes); eliciting responses from the groups, seeking any clarification (45 minutes); summarizing by the facilitator (15 minutes)	Listen to the lecture and take notes (60 minutes); solve the queries of the students (15 minutes)
After class	Again, go through the learning elements	Again, go through the learning elements
	Feedback questionnaire to be filled by students post-class test	

## Results

A total of 102 students participated in all the phases of the study, including the pre-class test, the flipped classroom, the traditional lecture, the post-class test and providing the feedback. Group A composed of 52 students and Group B contained 50 students.

There was no statistical difference between the two groups in the pre-class test.

The scores for the topic of cardiac output in the pre-class test for both the groups were the following: Group A=4.06 ±1.7, Group B= 3.76 ±1.46. The scores on the pre-class test for the topic, regulation of blood pressure, for both the groups were the following: Group A=3.73 ± 1.56, Group B=4.14± 1.68. Thus, the baseline knowledge of both groups on these topics was similar.

**Figure 1:**
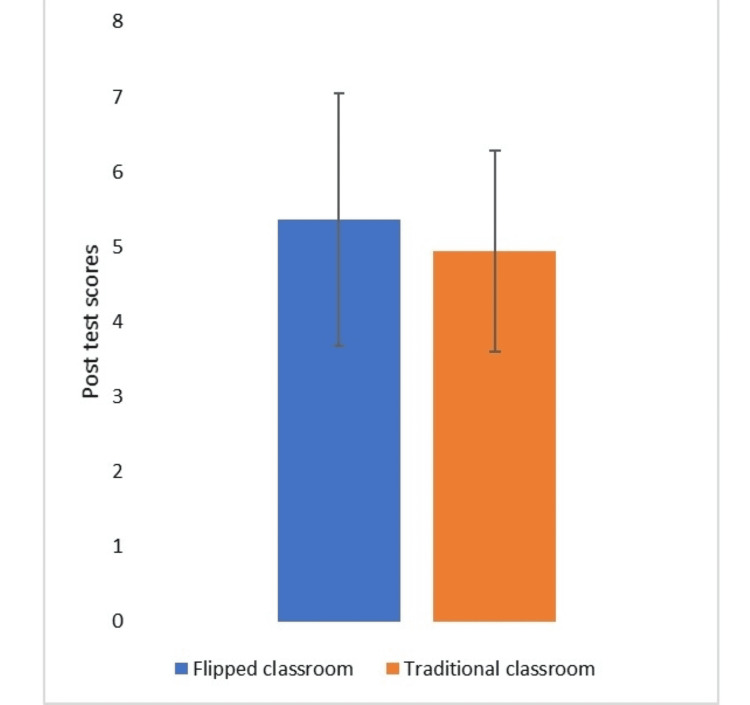
Comparison of students' post-test scores from different models Students were assessed with multiple-choice questions based on clinical vignettes after flipped classroom and traditional classroom sessions. An independent sample t-test was used to compare the differences between the flipped classroom and the traditional classroom. All values are presented as mean ± SD.

There was a statistically significant difference (p<0.05) between the post-test scores for the flipped classroom (5.36 ± 1.69) compared to the traditional method (4.94 ± 1.34) (Table 2, Figure 1).

**Table 2:** Comparison of the pre-test scores and post-test scores of the Flipped Classroom Group and the Traditional Lecture Group

	Flipped classroom (N=102)	Traditional classroom (N=102)	P value
Pre-test	4.1±1.67	3.75±1.9	0.1
Post-test	5.36±1.69	4.94±1.34	<0.05
P value	<0.01	<0.01	

The feedback of the students in both groups regarding the flipped classroom model is summarized in Table 3. The feedback collected responses regarding the learning experience, perceived value of the flipped classroom, the course materials, the teaching process and the evaluation system. Both the groups reported positively on all these aspects of the flipped classroom. The students strongly agreed that it was an enjoyable way of learning; it enhanced their problem solving and analytical ability; it developed their ability to work as a team member and plan their own work. The students also had a very positive perception of the teachers in this course. They strongly agreed that the teachers motivated them, provided them with enough feedback and were generally good at explaining things to them.

**Table 3:** Perception of the students regarding the flipped classroom Effect size is calculated by test statistic divided by the root of sample size (small effect: 0.1 <r ≤ 0.3, medium effect: 0.3 <r ≤0.5, large effect: r > 0.5). Both groups have similar feedback regarding flipped classroom approach as per the effect size.

	Group A (N=52)		Group B (N=50)				
	Mean	SD	Mean	SD	Z	P value	effect size
1. Questions regarding the learning experience							
The course met my expectations	3.48	0.51	3.35	0.66	-0.78	0.43	0.08
It is an enjoyable way of learning	3.54	0.59	3.57	0.58	-0.25	0.80	0.03
Overall, I am satisfied with the quality of the course	3.37	0.61	3.24	0.69	-0.80	0.42	0.08
The climate of this course is conducive to learning	3.24	0.64	3.35	0.72	-1.03	0.30	0.11
2. Questions regarding the value of the flipped classroom							
The flipped classroom has greatly enhanced my learning about this topic	3.41	0.65	3.35	0.72	-0.35	0.73	0.04
The flipped classroom course developed my problem-solving skills	3.20	0.69	3.39	0.64	-1.38	0.17	0.14
The flipped classroom course developed my analytical skills	3.35	0.60	3.27	0.76	-0.33	0.74	0.03
The flipped classroom course developed my ability to work as a team member	3.43	0.75	3.59	0.57	-0.88	0.38	0.09
As a result of the flipped classroom course, I feel confident about tackling unfamiliar problems	3.07	0.65	3.00	0.79	-0.31	0.76	0.03
The flipped classroom course developed my ability to work as a team	3.54	0.66	3.45	0.71	-0.70	0.49	0.07
The flipped classroom course developed my ability to plan my own work	3.15	0.76	3.10	0.80	-0.25	0.80	0.03
3. Questions regarding the course materials							
You usually have a clear idea of where you are going and what's expected of you in this course.	3.22	0.70	3.14	0.79	-0.39	0.70	0.04
It is always easy in this course to know the standard of work expected	3.11	0.80	3.14	0.76	-0.19	0.85	0.02
I was generally given enough time to understand the things we have to learn	3.24	0.79	3.41	0.70	-1.04	0.30	0.11
The work was too heavy	1.80	0.93	1.96	1.08	-0.54	0.59	0.06
The course is overly theoretical and abstract	2.20	0.96	2.33	0.97	-0.70	0.49	0.07
There was a lot of pressure on me to do well in this course	1.78	0.92	1.63	0.86	-0.83	0.41	0.09
The sheer volume of work to go through in this course means that you can't understand it all thoroughly	1.96	0.89	2.02	0.92	-0.32	0.75	0.03
4. Questions regarding the teaching process							
The staff on this course make it clear right from the beginning what they expect from the students	3.35	0.74	3.02	0.88	-1.88	0.06	0.19
The teachers on this course motivated me to do my best work	3.63	0.64	3.51	0.65	-1.17	0.24	0.12
The teacher's put a lot of time in commenting on student's work	3.26	0.80	3.10	1.03	-0.47	0.64	0.05
Teachers normally give helpful feedback on how you are doing	3.65	0.64	3.43	0.71	-1.85	0.06	0.19
Our lecturers are generally good at explaining things to us	3.46	0.66	3.27	0.84	-0.98	0.33	0.10
5. Questions regarding the evaluation system							
Teaching seem more interested in testing what you've memorized than what you have understood	2.96	1.05	3.06	1.07	-0.59	0.55	0.06
Too many staff on this course ask us questions just about facts	2.57	1.07	2.41	1.04	-0.73	0.47	0.07
To do well in this course all you need is a really good memory.	2.83	1.06	2.94	0.99	-0.46	0.65	0.05

## Discussion

To our knowledge, this is the first study to assess the acquisition of HOCS viz. application and analysis, using clinical vignette-based multiple-choice questions. The flipped classroom model was found to be superior to the traditional lecture model, especially regarding the acquisition of HOCS. The feedback provided by the students on the flipped classroom model suggests that this model is highly welcomed by the students.

Possible explanations may include:

Firstly, the flipped classroom model is student-centred. In the traditional lecture model, the locus of attention is on the teacher who delivers the course material to the students (‘sage on the stage’ approach). It is based on BF Skinner’s ‘instructivist’ (from the verb ‘instruction’) paradigm. Students are dictated in this learning endeavour. This occurs through lectures and textbooks. Thus, the students are only passive recipients of the information. The flipped classroom model is based on the ‘constructivist’ paradigm. This paradigm emphasizes the active and reflective nature of the learning experience. In the flipped classroom, the need to solve open-ended questions by watching pre-recorded lectures strongly motivates the students. This creates ‘strong chunks’ of information, just like laying bricks joined by solid mortar and building a strong wall. After watching the pre-recorded lectures, the students are encouraged to think critically and apply the knowledge they have gained. This process enables the creation of a new ‘cognitive schema’. This aspect fosters the acquisition of HOCS like application and analysis. As the student retrieves the information he has learnt during pre-recorded lectures, the memory traces are converted into long-term memory [10-12].

Secondly, the flipped classroom model personalizes the learning experience. The pre-recorded lectures provide students flexibility to decide when, where and how many times they are going to study in order to best fit their personalized learning style. Thus, this model enables ‘just-in-time’, ‘just-the-right-place’ and ‘just-the-right-time’ learning. Thus, this model enables individualization of the learning experience [8,13].

Thirdly, the flipped classroom model enables self-paced learning and fosters the attainment of ‘mastery.’ The concept of ‘mastery learning’ was briefly popular in the 1920s. However, it was revived by Benjamin Bloom in his paper ‘Learning for Mastery’ in 1968. The pre-class videos help the student to remember the facts and understand the concepts of a topic at his own pace. This frees up precious in-class time for the student to master the higher levels of Bloom’s Taxonomy viz. application and analysis [14].

Fourthly, today’s students are accustomed to turning to the web and social media for information and interaction. Thus, another purported benefit of the flipped classroom model is that 'it speaks the language of today’s students.' Another reason why the students find the lectures engaging is that the average attention span dips after 10 minutes. After 10 minutes have elapsed, students need to change the stimulus, and emotional variety or have an opportunity to step back and process what they are learning [15]. Thus, direct instruction can be broken down into more engaging, ten-minute bites of learning.

Finally, the flipped classroom model provides an opportunity for greater interaction between the teacher and students. This will enable the teacher to provide feedback to the student and immediately correct any misconceptions [16]. Such increased opportunities for feedback could improve student learning. This is because feedback has one of the strongest effect sizes of any instructional practice - in the 0.73 to 0.76 range according to two meta-analyses [17,18].

The main strength of the study was that it sought to assess the acquisition of HOCS like application and analysis. These were assessed by multiple-choice questions based on clinical vignettes. These vignettes were designed using the Blooming Biology Tool as a guide. This tool is a gold standard for designing assessments of various levels of Bloom's taxonomy. Ethical concerns mandated that the marks of the students will not be considered as part of the internal assessment. As 'assessment drives learning', the motivation of the students may be compromised as a result. The authors concede that this might be a potential weakness of the study.

## Conclusions

Taken together, the flipped classroom model was found to be beneficial for students’ learning. It enabled them to remember the facts and understand the concepts (both lower-order cognitive skills) during the before-class preparation, and used the valuable in-class time to master the application of the concepts and critically analyze them (application and analysis being higher-order cognitive skills). It provided the freedom and flexibility of self-paced learning, stimulated student interest, increased retention of the material and improved the skills of team-working. We believe that studies with larger student samples involving a greater number of topics will strengthen the evidence in favor of the flipped classroom. This model will prepare medical students with a better understanding of the course material, and foster the essential survival skills for a career in the twenty-first century.
